# Polyglycerol Ester-Based Low Energy Nanoemulsions with Red Raspberry Seed Oil and Fruit Extracts: Formulation Development toward Effective In Vitro/In Vivo Bioperformance

**DOI:** 10.3390/nano11010217

**Published:** 2021-01-15

**Authors:** Ana Gledovic, Aleksandra Janosevic Lezaic, Ines Nikolic, Marija Tasic-Kostov, Jelena Antic-Stankovic, Veljko Krstonosic, Danijela Randjelovic, Dragana Bozic, Dusan Ilic, Slobodanka Tamburic, Snezana Savic

**Affiliations:** 1 Department of Pharmaceutical Technology and Cosmetology, Faculty of Pharmacy, University of Belgrade, 11121 Belgrade, Serbia; ines.nikolic@pharmacy.bg.ac.rs; 2 Department of Physical Chemistry and Instrumental Methods, Faculty of Pharmacy, University of Belgrade, 11121 Belgrade, Serbia; aleksandra.janosevic@pharmacy.bg.ac.rs; 3 Department of Pharmacy, Faculty of Medicine, University of Nis, 18000 Nis, Serbia; marijatk@medfak.ni.ac.rs (M.T.-K.); dusanilicnis@yahoo.com (D.I.); 4 Department of Microbiology and Immunology, Faculty of Pharmacy, University of Belgrade, 11121 Belgrade, Serbia; jelena.stankovic@pharmacy.bg.ac.rs (J.A.-S.); dragana.bozic@pharmacy.bg.ac.rs (D.B.); 5 Department of Pharmacy, Faculty of Medicine, University of Novi Sad, 21000 Novi Sad, Serbia; veljko.krstonosic@mf.uns.ac.rs; 6 Department of Microelectronic Technologies, Institute of Chemistry, Technology and Metallurgy, University of Belgrade, 11000 Belgrade, Serbia; danijela@nanosys.ihtm.bg.ac.rs; 7 Cosmetic Science Research Group, London College of Fashion, University of the Arts London, London WC1V 7EY, UK; d.tamburic@fashion.arts.ac.uk

**Keywords:** nanoemulsion, phase inversion composition (PIC), biocompatible, low energy, polyglycerol ester, red raspberry seed oil, French oak fruit extract, antioxidant activity, MTT, skin hydration

## Abstract

This study focuses on the development of biocompatible oil-in-water (O/W) nanoemulsions based on polyglycerol esters, as promising carriers for natural actives: red raspberry seed oil—RO and hydro-glycolic fruit extracts from red raspberry—RE and French oak—FE. Nanoemulsions were obtained via phase inversion composition (PIC) method at room temperature by dilution of microemulsion phase, confirmed by visual appearance, percentage of transmittance, microscopic, rheological and differential scanning calorimetry (DSC) investigations. The results have shown that the basic RO-loaded formulation could be further enriched with hydro-glycolic fruit extracts from red raspberry or French oak, while keeping a semi-transparent appearance due to the fine droplet size (Z-ave: 50 to 70 nm, PDI value ≤ 0.1). The highest antioxidant activity (~92% inhibition of the DPPH radical) was achieved in the formulation containing both lipophilic (RO) and hydrophilic antioxidants (FE), due to their synergistic effect. The nanoemulsion carrier significantly increased the selective cytotoxic effect of RO towards malignant melanoma (Fem-X) cells, compared to normal human keratinocytes (HaCaT). In vivo study on human volunteers showed satisfactory safety profiles and significant improvement in skin hydration during 2 h after application for all nanoemulsions. Therefore, polyglycerol ester-based nanoemulsions can be promoted as effective carriers for red raspberry seed oil and/or hydro-glycolic fruit extracts in topical formulations intended for skin protection and hydration.

## 1. Introduction

Recently, natural raw materials obtained from the byproducts of the food and beverage industries have started to gain interest as promising ingredients in pharmaceutical and cosmetic applications. For example, red raspberry seed oil is recognized as a skin repair and anti-inflammatory active due to its high content of polyunsaturated fatty acids (PUFAs) and liposoluble vitamins (tocopherols and carotenoids) which are known to protect skin form free radical damage, acting also as UV-B and UV-A protectants [1,2,3,4]. Hydro-ethanolic extract from red raspberry fruit was shown to inhibit UVB-induced secretion of matrix metalloproteinase 1 and 3 and inflammatory factors IL-6 and IL-1β, while promoting type I procollagen synthesis, thus exhibiting anti-photoaging effects [5]. Other types of hydrophilic and lipophilic extracts obtained from berries and from wood waste products (such as oak acorns) also exhibit antioxidant and anti-carcinogenic activity [6,7,8,9,10]. The above-mentioned extracts are rich in bioactives which are usually sensitive to heat, light and air, thus requiring effective carriers and mild processing conditions to preserve their activity.

Nanonization has emerged as a promising strategy for enhancing viability of natural ingredients in advanced pharmaceutical and cosmetic products, with nanoemulsions being a particularly promising filed of research [11,12,13]. Nanoemulsions represent submicron colloidal systems, i.e., transparent or translucent emulsions with very fine droplet sizes (preferably up to 100 nm, but can be up to 300 nm) which are kinetically stable and have an improved resistance to gravity-induced instability phenomena (e.g., creaming or sedimentation) and prolonged shelf life compared to classical emulsions [14,15]. Compared to microemulsions (transparent and isotropic thermodynamically stable nanosystems also bellow 100 nm), which can be prepared from similar components as nanoemulsions (surfactants, cosurfactants, cosolvents, oil phase and water), nanoemulsions are formed with lower total surfactant-to-emulsion ratio (SER), usually up to 15, and lower surfactant-to-oil ratio (SOR), usually 1 to 2. Thus, nanoemulsions of the oil-in-water (O/W) type are considered more favorable for skin application due to higher water content, lower skin irritation potential and commercial reasons [12,13,16]. Other desirable nanoemulsion features well-suited for topical formulations include pleasant visual appearance, high fluidity and good spreadability on skin surface, while the large surface area of small nanodroplets on the skin leads to improved or controlled penetration of active molecules [9,17].

Along with the nanonization approach, the growing demand from the consumers pushes the industry towards the development of new environmentally friendly, nontoxic, biodegradable surfactants aiming at “green” formulations. One particularly promising class of natural surfactants suitable for pharmaceutical, cosmetic and food industries are polyglycerol esters of fatty acids. These non-ionic natural surfactants are obtained by esterification of natural glycerol, as the hydrophilic part of a surfactant molecule, with fatty acids from different natural plant oils as its lipophilic domains. Depending on the number of glycerol units with different degrees of esterification and the properties of fatty acids (e.g., chain length and unsaturation), various polyglycerol esters and their mixtures can be obtained [18,19]. Apart from some patents with limited information [20,21], only a few reports are available regarding the formation of O/W nanoemulsions or microemulsions with polyglycerol esters via the low energy methods [22,23,24,25]. Therefore, most of these novel surfactants have not yet been characterized or compared with the traditionally used polyethoxylated surfactants (such as Polysorbates). It should be noted that the bio-origin does not automatically lead to a safe and effective ingredient, thus all natural raw materials and the corresponding formulations have to be thoroughly assessed [17].

Based on the above, the aim of this study was to develop and evaluate the nanoemulsion formation with polyglycerol esters using low-energy phase inversion composition (PIC) method, as a potential natural nanocarrier system for lipophilic antioxidant (red raspberry seed oil) and/or hydrophilic antioxidants (from red raspberry and French oak fruits). This study presents the continuation of the previously published work on the Poly-sorbate 80-based nanoemulsions [9]. Different in vitro and in vivo tests were performed in order to assess the biological activity and safety of the obtained nanoemulsions. In vitro antioxidant activity was checked via DPPH assay, to ensure that bioactivity is preserved in nanoemulsions during storage. In vitro cytotoxicity tests were performed on healthy human skin cells (keratinocytes) and malignant melanoma cells (Fem-X), to gain insight on the selectivity of formulations towards skin cancer cells. Finally, the optimized nanoemulsions were tested on human volunteers, aiming to assess their safety and effectiveness as skin moisturizers, as an important factor to maintain skin health and function. Therefore, the current study aimed at deeper understanding of the functionality and safety of polyglycerol esters as stabilizers for different natural raw materials on a nanoscale, which should lead to the design of new, healthier, and more efficient products suitable for multiple industries.

## 2. Materials and Methods

### 2.1. Materials

The main surfactant polyglyceryl 4-laurate (P4L) (Tego Care^®^ PL4) was mixed with a surfactant blend (TS)—polyglyceryl-6 caprylate, polyglyceryl-3 cocoate, polyglyceryl-4 caprate, polyglyceryl-6 ricinoleate (1:1:1:1) (Tego Solve^®^ 61, Evonik Nutrition and Care GmbH, Essen, Germany). Preservative mixture—phenoxyethanol, ethylhexylglycerin (Euxyl PE^®^ 9010), produced by Schülke GmbH, Norderstedt, Germany, and red raspberry fragrance oil, produced by Jean Products, Karsbach, Germany, were tested as potential cosurfactants.

The oil phase Ethylhexyl pelargonate (EP) (Crodamol^®^ OPG) was received as a free sample from Croda, East Yorkshire, UK. Red raspberry cold-pressed, unrefined, organic seed oil (INCI name: Rubus idaeus seed oil) was obtained from domestic company Terra Co, Novi Sad, Serbia, and it was produced by Aromaaz International, New Delhi, India.

Water phase: Ultra-purified water was obtained with GenPure apparatus (TKA Wasseranfbereitungssysteme GmbH, Neiderelbert, Germany). Additional water phase additives included Red raspberry fruit extract—Fruitliquid^®^ raspberry (INCI: water, propylene glycol, Rubus idaeus fruit extract) and French oak fruit extract—Phytessence^®^ French oak (INCI: Aqua, Glycerin, Quercus petraea fruit extract), received as free samples from Crodarom, Chanac, France. Glycerol (HPLC grade) was produced by Fisher Chemical, Waltham, ME, USA. The pH of the nanoemulsion was adjusted with freshly prepared 0.1 M sodium hydroxide aqueous solution.

Reagents for the DPPH assay: 2,2-Diphenyl-1-picrylhydrazyl (DPPH), 3,4,5-trihydroxybenzoic acid (gallic acid) and methanol (HPLC grade) were produced by Sigma-Aldrich (St. Louis, MO, USA).

### 2.2. Methods

#### 2.2.1. Nanoemulsion Preparation and Characterization

##### Phase Inversion Composition (PIC) Method

Nanoemulsions were prepared according to the phase inversion composition (PIC) method [9,15] by stepwise addition of the water phase to the organic phase (surfactants/cosurfactant and oils) under continuous vortex mixing at 1300 rpm, to obtain a maximum sample volume of 5 to 10 mL. During the optimization phase, the following components were mixed at various ratios: surfactants (P4L and TS), oil phase (EP, red raspberry seed oil and cosurfactants) and water phase (water, glycerol) to detect the minimal SOR and SER necessary to form nanoemulsions with mean droplet sizes < 100 nm and narrow polydispersity index (PDI < 0.15). The transient phases occurring during the nanoemulsion formation were detected by visual, microscopic and rheological methods. Additionally, some nanoemulsions were loaded with antioxidant hydro-glycolic extracts from red raspberry or French oak fruit to investigate their influence on nanoemulsion stability and in vitro/in vivo performance. The pH value of the samples was adjusted with 0.1 M NaOH solution, to obtain pH values suitable for the application on human skin (pH ~3.5 to 5). Nanoemulsion samples were stored at the room temperature for 3 months, and the parameters relative to the nanoemulsions’ physicochemical stability (droplet size, PDI, pH value and electrical conductivity) were checked monthly.

##### Particle Size Distribution

The mean droplet size (intensity weighted mean diameter, Z-average diameter—Z-Ave) and droplet size distribution (PDI) of the nanoemulsions were determined by photon correlation spectroscopy (PCS), using Zetasizer Nano ZS90 (Malvern Instruments Ltd., Worcestershire, UK). Before measurement, each nanoemulsion sample was diluted with ultra-pure water (1:100, v/v). To investigate the presence of larger particles and aggregates, a laser diffraction technique (LD) was applied using Malvern Mastersizer 2000 (Malvern Instruments Ltd., Worcestershire, UK), whereby the volume weighted diameters d50, d90 and D [3,4] were obtained as critical sizing parameters. The samples were measured 24 to 48 h after production and the measurements were repeated at the predefined intervals, during the 3-month storage at the room temperature.

##### Microscopic Investigations

(a) Polarized light microscopy

Optical microscopy under polarized light was employed to investigate the signs of anisotropy in the transient phases and nanoemulsion samples (×100 magnification) with Olympus BX53P polarizing microscope, and the obtained images were analyzed with the cellSens Entry version 1.14 software (Olympus, Tokyo, Japan).

(b) Atomic force microscopy

To gain a direct access into the microstructure, i.e., to determine its morphological properties and to confirm the mean droplet size data, the representative nanoemulsion samples F1 and F2 prepared with different cosurfactants were investigated using Ntegra prima atomic force microscope (NT-MDT). Prior to measurements, 10 μL of diluted nanoemulsions (1:100 or 1:1000 v/v) were placed on the circular mica substrate discs (Highest Grade V1 AFM Mica Discs, Ted Pella Inc., Redding, CA, USA) and dried under vacuum for 24 h at 25 °C. Measurements were carried out in the air using intermittent-contact AFM mode. For this purpose, NT-MDT NSGO1 silicon cantilevers (N-type, Antimony doped, Au reflective coating) were used. The nominal force constant of these cantilevers was 5.1 N/m. During the measurements cantilever driving frequency was around 150 kHz. Both topography and “error signal” AFM images were taken, and later analyzed using Image Analysis 2.2.0 (NT-MDT) software.

##### Spectroscopic Characterization of Optical Clarity

Optical clarity of the transient phases (containing 30 or 50 wt% water phase) and the corresponding nanoemulsions (containing 80 wt% water phase) was checked spectroscopically with Evolution 300 UV-VIS spectrophotometer (Thermo Scientific, Loughborough, UK) by measuring the percentage of transmittance (%TP) of the samples at 600 nm, using ultra-pure water as a blank.

##### Electrical Conductivity and pH Value Measurements

Electrical conductivity was measured with Sension+ EC71 apparatus (HACH, Loveland, CO, USA) during the titration of pure surfactant mix with water phase or directly during the nanoemulsion formation by water titration of the organic phase (containing oil and surfactant mix). This was based on the fact that electrical conductivity values can provide preliminary insight into the internal structure of the sample and detect the phase transition point. The pH values of the obtained nanoemulsions were measured in undiluted samples using HI9321 microprocessor pH meter (Hanna Instruments Inc., Ann Arbor, MI, USA). The measurements were performed in triplicate at 25 ± 2 °C.

##### Differential Scanning Calorimetry

Differential scanning calorimetry (DSC) investigation of water freezing behavior of the samples was performed using Mettler Toledo DSC 1, STARe System (Mettler Toledo GmbH Analytical, Giessen, Germany). In order to investigate the structure of the transient phases (containing 30 and 50 wt% water phase) and the final nanoemulsions (containing up to 80 wt% water phase), approximately 10 mg of the sample was placed in aluminum pans and hermetically sealed. DSC curves were generated by cooling the samples from −25 to −60 °C, at the cooling rate of 5 K/min. An empty sealed aluminum pan was used as a reference.

##### Continuous Flow (Hysteresis Loop) Test

The determination of rheological behavior of the transient phases and the corresponding nanoemulsions was carried out by the rotational viscometer HAAKE Mars rheometer (“Thermo Electron Corporation”, Karlsruhe, Germany) at the constant temperature of 25 ± 0.1 °C. Continuous flow tests (hysteresis loop tests) were carried out with a cylinder DG 41 Ti sensor. The test consisted of three stages: 120 s of increasing shear rate from 0.5 to 100 s^−1^, followed by 60 s of constant shear rate of 100 s^−1^ and finally 120 s of decreasing shear rate back to 0.5 s^−1^. In total, three measurements were performed for each tested sample. Consistency and flow indices were determined from the power law presented in Equation (1) for quantitative analysis of flow behavior:

(1) $$\tau =k\;\times \;{\dot{\gamma }}^{n}$$

where *τ* represents shear stress, $\dot{\gamma }$—shear rate, *k*—consistency index and *n*—flow index.

#### 2.2.2. In Vitro Antioxidant Activity—The DPPH Assay

The DPPH assay in methanol was performed similarly to our previous work [9], with small modifications. Nanoemulsions were tested at several concentrations (10, 30 and 50 µL/mL), while the nanoemulsion solutions in methanol served as blanks. Results were expressed as % INH (percentage of DPPH inhibition) calculated according to Equation (2):

% Inhibition of DPPH radical = [(A_DPPH_ − A_sample_)/A_DPPH_] × 100 (2)

where A_DPPH_ is the absorbance of the DPPH standard sample (5 mL of DPPH standard solution mixed with 5 mL methanol) and A_sample_ is the absorbance of a sample after 30 min. Gallic acid was used as a standard antioxidant to validate the method.

#### 2.2.3. In Vitro Cytotoxic Activity

##### Preparation of Stock Solutions

The stock solutions of lipophilic raw materials red raspberry seed oil (RO) and red raspberry fragrance oil (RF) were firstly prepared in dimethyl sulfoxide (DMSO), and then diluted with the nutrient medium so that the final DMSO concentration was below 0.1% and the relevant working concentrations of 12.5–400 mcg/mL were obtained. The hydro-glycolic extracts from RE and FE and the RO-loaded nanoemulsions were diluted directly with the medium to the relevant working concentrations (12.5–400 mcg/mL of pure extract or calculated as active ingredient in nanoemulsions). The placebo nanoemulsion, without RO or hydro-glycolic antioxidant fruit extracts, was diluted in the same manner as the active samples. For all the cells used, the nutrient medium was RPMI-1640 with HEPES (25 mM) (Sigma-Aldrich, St. Louis, MO, USA) supplemented to final concentration with L-glutamine (3 mM), streptomycin (100 µg/mL), penicillin (100 IU/mL) and fetal bovine serum (10%; FBS; 56 °C heat inactivated due to inactivation of cholinesterases and system complement), adjusted to pH 7.2 (bicarbonate solution).

##### Cell Cultures

The Fem-X (human malignant melanoma cells) and non-tumorous HaCaT (human keratinocyte cells) were cultured as monolayers in the nutrient medium. All of these cells were grown at 37 °C in 5% CO_2_ and a humidified air atmosphere. The Fem-X cells were seeded as 2 × 10^3^ cells per well/100 µL, while HaCaT cells were seeded as 5 × 10^3^ cells per well/100 µL, into 96-well microtiter plates. After the cell adherence, 20 h later, different concentrations of the test samples (corresponding to final concentrations of 12.5 mcg/mL to 400 mcg/mL of the lipophilic or hydrophilic extracts) were added to the wells.

##### Determination of the Target-Cell Survival

The cell survival was determined by the MTT test 48 h after the addition of test samples, according to the method of Mosmann [26], with slight modifications. Briefly, 10 μL of MTT (3-(4,5-dimethylthiazol-2-yl)-2,5-diphenyl tetrazolium bromide, Sigma-Aldrich, St. Louis, MO, USA) solution in phosphate buffered saline (pH 7.2, 5 mg/mL) was added to each well. The samples were incubated for an additional 4 h at 37 °C in 5% CO_2_ and humidified atmosphere. Afterwards, 100 μL of 10% sodium dodecylsulfate (Sigma-Aldrich, St. Louis, MO, USA) was added to each well, and the absorbance of the cell medium from each well was measured at 570 nm the next day using a micro plate spectrophotometer (Multiscan™ FC Microplate reader, Thermo Fisher, Waltham, MA, USA). To calculate the cell survival (%), the absorbance at 570 nm of each of the samples with the cells grown in the presence of the test compounds were divided by the absorbance of the control sample (the absorbance of the cells grown in nutrient medium only), after the subtraction of the blank sample absorbance. The IC50 values (the concentration of the compound which decreased the survival of treated cells by 50%) were determined from the graph by numerical analysis of the obtained data. Three independent experiments were performed, each sample in triplicate, and results were presented as mean values ± SD.

#### 2.2.4. In Vivo Safety and Efficacy Assessment

##### Safety Profile

Safety profiles of the optimized nanoemulsion samples were evaluated in vivo by measuring relevant biophysical human skin parameters: transepidermal water loss (TEWL), erythema index (EI) and stratum corneum hydration (SCH) upon cessation of 24 h occlusive treatment. Since the increase in TEWL is observed after the application of skin irritants, TEWL measurements are often used in support of cosmetic claims of product mildness [27]. All skin parameters were measured at the start (baseline values on the first day of the experiment) and 60 min upon cessation of a 24 h occlusive treatment (on the second day of the experiment). A total of 12 healthy volunteers (average age 26.3 ± 5.1) were recruited. The flexor side of the right forearm was treated with the samples F1 and F2, using precisely delineated cardboard template (with three empty rectangular spaces, 16 cm^2^ each). The third site served as a non-treated control without occlusion (UC). Similarly, the flexor side of the left forearm was treated with the samples F1 RE and F1 FE, while the third site was left as a non-treated control under occlusion (UCO). The amount of 0.016 g/cm^2^ of the sample was applied to a respective skin site, spread vigorously with a rubber-gloved finger, immediately covered with Parafilm^®^, then with cotton adhesive tapes and left for 24 h.

##### Moisturizing Efficacy and Skin pH Value

The in vivo moisturizing efficacy was investigated in a short-term (2 h) study measuring SCH. The influence of nanoemulsions on the skin pH value was also checked during the first 2 h, as another parameter relevant for their safety assessment [12,28]. To estimate the moisturizing efficacy and the skin pH value, additional group of 11 healthy volunteers (23.2 ± 1.6 years) was recruited. The flexor side of the right forearm was treated with the samples F1 and F2 using precisely delineated cardboard template (with empty rectangular spaces, 16 cm^2^ each). The flexor side of the left forearm was treated with the F1 5% RE and F1 5% FE. One rectangle was left as an untreated control (UC). The amount of sample applied on skin surface was 0.016 g/cm^2^, and the following measurements were conducted at different time intervals: baseline (before the application of the samples), 0.5, 1 and 2 h after application. All parameters were measured according to the published guidelines [27,29]. In vivo measurements were performed in accordance with the Declaration of Helsinki, whereby the volunteers were thoroughly informed of the study before signing a written consent. The study was approved by the Ethical Committee on Human Research of the Faculty of Medicine, University of Nis, Serbia. Participants were instructed not to use any skin cleansing or skin care products on the test sites during the week prior to the study, as well as throughout the experiment. All had healthy skin and no known allergy to any ingredient in the samples. Before any measurements were taken, participants were asked to acclimatize for 30 min under controlled conditions (21 ± 1 °C and 50 ± 5% RH). TEWL was measured using Tewameter^®^TM 210, EI using Mexameter^®^MX 18, SCH by means of Corneometer^®^CM 825, and skin pH using pH meter 900 (Multi Probe Adapter System MPA9), all instruments by Courage and Khazaka, Koln, Germany.

### 2.3. Statistical Analysis

In vivo skin performance data were presented as means +/− standard error of the means (SEM). Measured parameters (SCH, TEWL and EI) were expressed as absolute changes to baseline (Δ values of the second versus the first day) in the safety study. In vivo effects of the samples were compared to each other and to their respective controls, under and without occlusion (UC and UCO), using the one-way ANOVA, followed by Tukey’s Honest Significance Test (HST) where appropriate. In the moisturizing efficacy test, the SCH was presented as an absolute change compared to baseline for both test samples and UC. The data from different sites were analyzed using one-way ANOVA, followed by Tukey’s HST, where appropriate. The SCH and skin pH values for the same sample at different time points were compared using paired *t*-test. The differences were accepted as statistically significant at *p* < 0.05. Statistical analysis was performed with commercial statistical software SPSS for Windows 17.0. All other data are represented as mean values +/− standard deviation.

## 3. Results and Discussion

### 3.1. The Optimization of Nanoemulsion Formulations

Among the available nanoemulsion production methods, the low energy methods performed with minimal energy input (e.g., Phase inversion composition method at room temperature) are highly suitable for natural and thermo-sensitive molecules [9,15]. Methods that are based on the physico-chemical bottom-up approach rely on the release of chemical energy when carefully selected ingredients are mixed in a specific way, leading to surfactant self-assembly and spontaneous formation of small nanodroplets [14,15,30]. In order to optimize the formulation, the surfactant, cosurfactant, oil and water phase properties and their mutual ratios must be thoroughly investigated, because these composition factors play a crucial role in the formation and final characteristics of low-energy nanoemulsions [14,30].

#### 3.1.1. The Role of Polyglycerol Ester-Based Surfactants

In this study, naturally-derived biocompatible polyglycerol ester-based surfactants were investigated, aiming to replace non-ionic polyethoxylated surfactants (i.e., Polysorbates) that are widely employed in submicron formulations with plant oils and lipophilic vitamins [31,32]. Polyglycerol-4 laurate (P4L) was chosen as the main surfactant, based on the previous reports involving its suitability for topical microemulsions [22] and nanoemulsions [23]. In order to obtain a “natural label-friendly” formulation, a novel emollient oil derived from plant raw materials, ethylhexyl pelargonate (EP), was used. EP has shown very good spreadability, light skin feel and good safety profile in topical formulations [33], but it has been under-explored in nanoemulsions, where it could potentially replace traditionally used medium chain triglycerides.

This study has revealed that P4L did not form nanoemulsions with EP as a single oil phase component. P4L had to be combined with the additional polyglycerol ester mixture TS in order to obtain nanoemulsion. This finding is in line with the previous studies [22,23,24,25], where mixed polyglycerol ester-based surfactants, as well as several oil phase additives and/or polyols, were employed to obtain stable micro/nanoemulsions. Thus, TS which is derived from several different fatty acids, is expected to enhance the solubilization capacity of the P4L/TS surfactant mix for different types of oils. Among the several tested P4L: TS ratios, 1:1 ratio was found to be the optimal one, which could be linked to an optimal viscosity of the mixed surfactant system, which promoted the formation of nanoemulsions via the chosen PIC method. TS is a clear liquid with low viscosity at 25 °C, whereas P4L is a cloudy and viscous paste; therefore, the addition of TS to P4L reduces the viscosity of the surfactant mix and facilitates its mixing with the oil phase [34]. However, it was found that the nanoemulsion region was very narrow and that the minimal SER and SOR values resulting in the formation of nanoemulsion with EP as a carrier oil were 10 and 1, respectively (droplet size ~187 nm, PDI ~0.35). Therefore, the above minimal SOR and SER values were used for all further oil and water phase optimization. The higher SOR/SER values resulted in a formation of bigger nanodroplets, more polydisperse or even two-phase Winsor systems.

#### 3.1.2. The Role of Cosurfactants, Cosolvents and Red Raspberry Seed Oil

In order to decrease the droplet size of the basic formulation with EP (containing 10 wt% P4L: TS surfactant mix, 10 wt% EP and 80 wt% water), several potential cosurfactants were added to the nanoemulsion oil phase, prior to the titration with water. The addition of a very small amount (0.5 wt%) of a preservative mixture (consisting of phenoxyethanol and ethylhexylglycerin), or the addition of raspberry fragrance oil (2 wt%) resulted in a dramatic decrease of droplet sizes (~132 nm, PDI: ~0.23, and ~89.6 nm, PDI: 0.07, for the preservative or fragrance, respectively). However, the combination of these two cosurfactants or higher concentrations of each individual cosurfactant increased droplet sizes, causing system instability (Table S1, Supplementary file). Our findings are in line with a study by Heunemann et al. [23] that reported phenoxyethanol as a very efficient cosurfactant for P4L in the nanoemulsion formation via the PIC method. In our study, raspberry fragrance oil also acted as a cosurfactant, which is a behavior observed for some essentials oils, due to their high content of phenolic and other alcoholic compounds that can be positioned at the oil-water interface [13,35]. These findings have a practical formulation value, since the role of preservative or fragrance in nanoemulsion formation and stability has been neglected in most research papers, despite the fact that additives are readily used in pharmaceutical/cosmetic industry.

The incorporation of plant oils into nanosystems with very fine droplets (<100 nm) is a challenging task because of the oils’ complex composition, i.e., the presence of bulky fatty acid tails in triglyceride molecules [36,37]. It was found in this study that a relatively small amount (2 wt% of red raspberry seed oil—RO, out of 10 wt% total oil phase), was the optimal concentration to use, while higher RO concentrations have produced milky, unstable emulsions. Importantly, it was necessary to add a cosolvent glycerol (at the optimal concentration of 30 wt%, relative to the water phase) to obtain transparent/translucent nanosystems containing RO, regardless of the cosurfactant used in the oil phase. This optimized water phase composition led simultaneously to the formation of very fine nanodroplets (<65 nm, PDI < 0.1) and improved nanoemulsion stability (Table S2, Supplementary file).

The suitability of glycerol as a cosolvent for polyglycerol-ester based systems was somewhat expected, because of the presence of glycerol groups in the surfactant mix and in the triglycerides in plant oils. It is known that the addition of glycerol leads to the changes of polarity and viscosity of the water phase, which in turn facilitates the formation of microemulsions [36,37]. Polyols have also been reported to promote the formation of low-energy nanoemulsions and microemulsions by their direct interaction with the surfactant molecules at the oil-water interface, leading to the change in surfactant curvature [25,38].

### 3.2. Characterization of the Transient Phases during the PIC Nanoemulsion Formation and the Final Nanoemulsions

#### 3.2.1. PIC Mechanism of Nanoemulsion Formation

**Figure 1:**
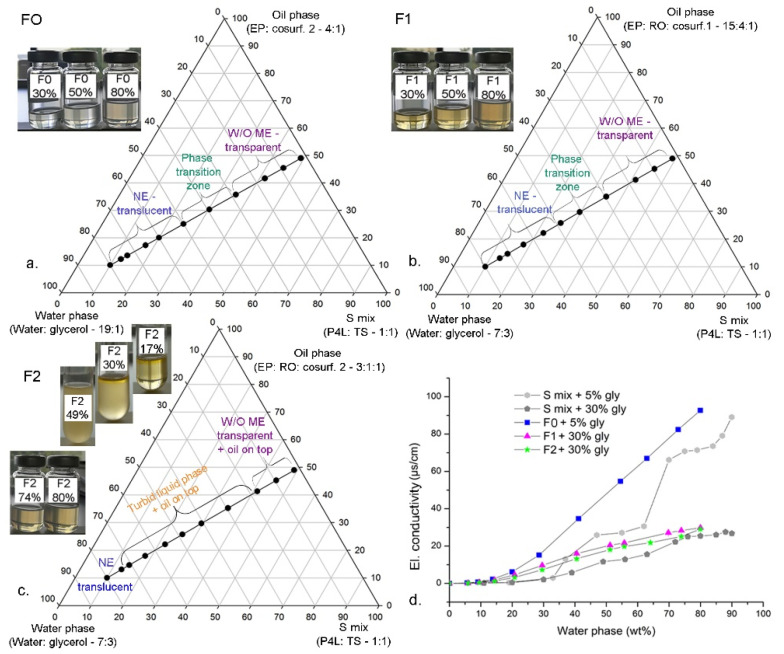
Visual appearance and phase transitions as a function of water phase content along the Line 5 (surfactant-to-oil ratio—SOR = 1) optimal for the nanoemulsion formation: (**a**) F0 samples prepared with cosurfactant 2, without red raspberry seed oil (RO); (**b**) F1 samples prepared with cosurfactant 1 and RO; (**c**) F2 samples prepared with cosurfactant 2 and RO; (**d**) Electrical conductivity curves as a function of the water phase content of the polyglycerol ester-based surfactant mix (P4L:TS = 1:1)/water phase (glycerol/water) binary mixtures and the corresponding nanoformulations (F0, F1 and F2) formed via the phase inversion composition (PIC) method. ME—microemulsion, NE—nanoemulsion.

In the applied PIC production method performed by a stepwise addition of the water phase to the appropriate surfactant/cosurfactant/oil mix, the surfactant-oil-water (SOW) system passes through one of the several possible transient phases (e.g., liquid crystalline (LC) phase/microemulsion (ME)/oil-in-water-in-oil (O/W/O) multiple emulsion) prior to its conversion to O/W nanoemulsion [9,14,15]. Therefore, it was crucial to reveal the nanoemulsification pathways that lead to the nanoemulsions with the desired characteristics of small droplet sizes with narrow distribution and prolonged stability [25]. Based on the results of the formulation optimization study, several samples of the detected transient phases and nanoemulsions containing different cosurfactants, oil and water phases were prepared and characterized (Figure 1, Table 1).

During the addition of water phase, depending on the system composition, two distinct nanoemulsification pathways were identified. In the first case, the transparent or translucent transient isotropic phases were observed for up to ~50 wt% water phase in the systems prepared with phenoxyethanol and ethylhexyl glycerol (cosurfactant 1) with EP as carrier and RO in the oil phase (F1 samples, with 30 wt% and 50 wt% water phase). Similar behavior was observed in the samples prepared with raspberry fragrance oil—RF (cosurfactant 2) and EP (F0 samples, with 30 and 50 wt% water phase) without RO (Figure 1a,b). All transient phases and the corresponding nanoemulsions containing 80 wt% water phase exhibited Newtonian flow behavior (with n-value ~1), indicating a microemulsion phase which is latter converted into O/W nanoemulsion (Table 1, Figure S1, Supplementary file). In line with that, the % TP decreased as the water content increased, indicating that a transparent microemulsion phase is converted by dilution into a translucent nanoemulsion after the critical concentration of water phase is reached (at ~40 to 50 wt%) (Table 1). The second nanoemulsification pathway was observed when RO was present in the oil phase and cosurfactant 2 (RF) was used, which included a two-phase system (transparent microemulsion and a wide turbid liquid phase, both with excess oil on top). Interestingly, the transition into a translucent O/W nanoemulsion appeared at a much higher water content (at ~74 wt%) (Figure 1c).

Additional confirmation of the structural arrangements was obtained by electrical conductivity measurements during water titration process and by a thermal technique (DSC), which can detect water freezing behavior and its location within the SOW system [39,40]. Electrical conductivity values were very low (<10 µS/cm) for transient phases with up to ~20 wt% water phase, which indicated the presence of water-in-oil (W/O) ME (Figure 1d). With further water addition, a gradual increase (in samples F1 and F2, with high glycerol content) or a sharper increase in conductivity (in samples F0, with small glycerol content) was observed, which can be ascribed to the occurrence of water channels in the bicontinuous microemulsion [40,41]. As the water phase content reached critical value, the transient structure was disrupted, forming O/W nanoemulsion and the conductivity gradually increased with the addition of the remaining water (Figure 1d). Electrical conductivity remained low (≤30 µS/cm) in all samples prepared with high glycerol content (30 wt% relative to the water phase), confirming that surfactant molecules in surfactant/water/glycerol mixtures (S mix) or in O/W nanoemulsion (F1 and F2) remained highly associated with the molecules of glycerol.

**Figure 2:**
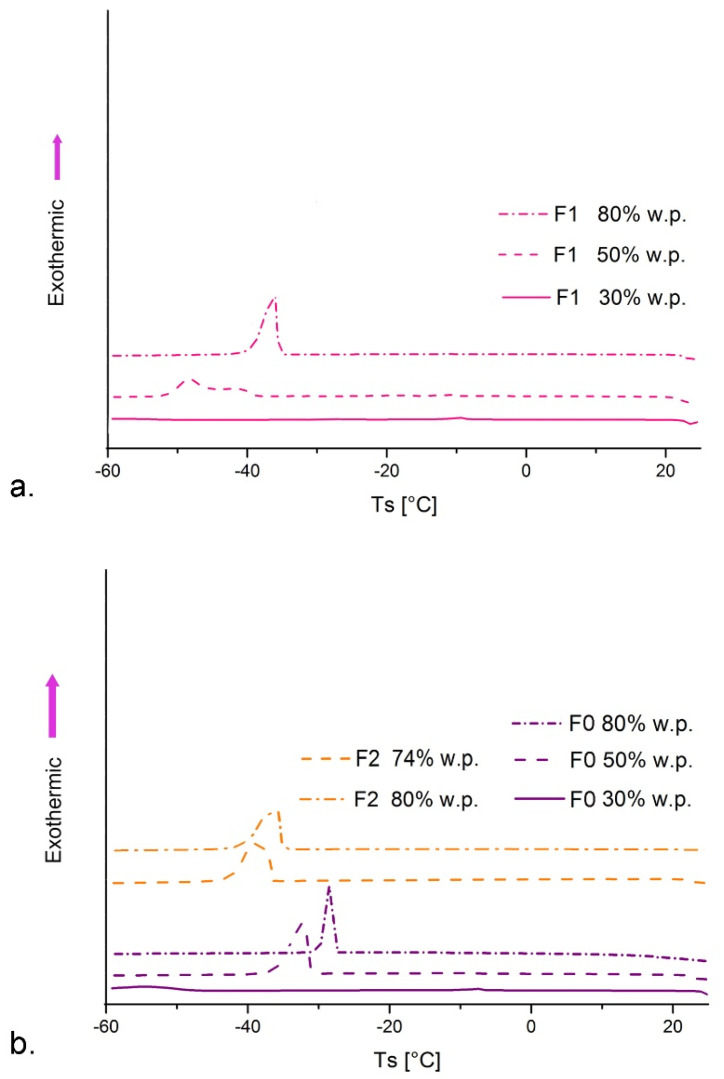
Differential scanning calorimetry (DSC) curves in cooling mode: (**a**) formulations F1 (with cosurfactant 1) and (**b**) formulations F0 and F2 (with cosurfactant 2) with different water phase content (wt%), 7 days after preparation.

DSC curves of the transient phases prepared at the critical water phase concentrations (30 and 50 wt%) and DCS curves of the final nanoemulsions (containing 80 wt% water phase) (Figure 2) imply that the structural transitions during the nanoemulsion formation process occurred as follows: W/O microemulsion/bicontinuous microemulsion (phase transition zone)/O/W nanoemulsion. In the samples with low water content (e.g., 30 wt% water phase) the absence of the water peek was apparent, which can be ascribed to W/O microemulsion with non-freezable bound water. As the water content increased (at 50 wt% water phase) the phase transition takes place and the water peak started to appear, but shifted to the lower freezing temperatures (around −30 to −50 °C) compared to pure water (usually around 0 to −15 °C), due to the interactions with surfactant molecules and polyols [39,40]. At 80 wt% water phase, the water peak was prominent because water became the continuous phase in O/W nanoemulsion and the most abundant component of the system. These findings are in line with previously reported thermal behavior in the cooling mode for nanoemulsion [39] and microemulsion systems [40]. The shift towards lower temperatures occurred in the final nanoemulsions with RO containing 30 wt% glycerol (relative to the nanoemulsion water phase), due to strong cryoprotective effect of glycerol, the behavior previously reported for systems containing high amounts of polyols [37,40].

#### 3.2.2. Microscopic Investigations

**Figure 3:**
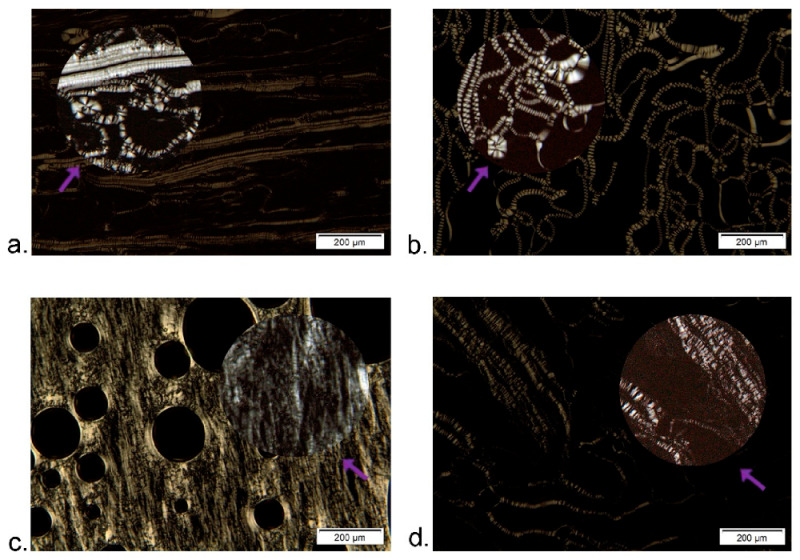
Optical textures under polarized light of the polyglycerol ester-based surfactant mix (S mix = P4L: TS 1:1)/water phase (glycerol/water) binary mixtures: (**a**) S mix 70 wt%, 30 wt% (5 wt% glycerol in water) and (**b**) S mix 46 wt%, 54 wt% water phase (5 wt% glycerol in water) exhibit lamellar phase with oily streaks and Maltese crosses and optically isotropic regions (dark); (**c**) S mix 70 wt%, 30 wt% (30 wt% glycerol in water) exhibits hexagonal phase and air bubbles—dark circles (**d**) S mix 46 wt%, 54 wt% water phase (30 wt% glycerol in water) exhibits hexagonal phase (bright) and isotropic domains (dark). Micrographs were taken at 100 magnification, and the close-up of the prominent anisotropic structures are marked with purple arrows (up to 400× magnification).

The isotropic behavior was confirmed for the microemulsion transient phases and the resulting nanoemulsions (the field of view remained dark). In contrast to that, the poly-glycerol ester-based surfactant mix used in our study formed anisotropic liquid crystalline structures of lamellar (Figure 3a,b) or hexagonal type (Figure 3c,d) during the titration with the water phase (5 and 30 wt% glycerol solution, respectively), which is a known behavior for some surfactants at higher concentrations [42,43]. In fact, it was previously reported that polyglycerol ester-based surfactant obtained from rice bran oil forms lamellar structures in water [18]. Wakisaka et al. [24] reported the formation of anisotropic liquid crystalline phases with polyglycerol-ester surfactants during the nanoemulsion formation. However, our investigation revealed that the microemulsion (isotropic, transparent liquid phase) was formed as a nanoemulsion precursor, which is in line with previous reports [15,23].

**Figure 4:**
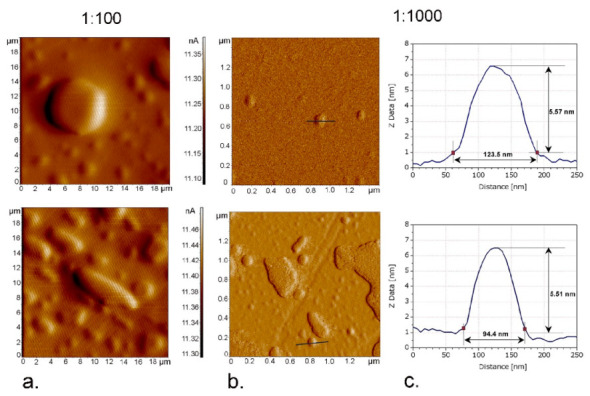
Atomic force microscopy (AFM) of the selected nanoemulsion samples F1 (upper row) and F2 (lower row) at different dilutions: (**a**) 2D “error signal” of 20 × 20 µm^2^ of the samples at 1:100 dilution (**b**) 2D “error signal” of 2 × 2 µm^2^ of the samples at 1:1000 dilution with marked droplet profile; (**c**) height profiles of the two selected nanoemulsion droplets at 1:1000 dilution.

AFM analysis revealed the tendency of diluted nanoemulsions to form aggregates during drying process (Figure 4). At 1:100 dilution of nanoemulsions, the presence of large round, oval and “island-like” aggregates were observed, due to the gel formation which was in line with the findings of Alessandrini and Facci [44]. At higher dilution (1:1000) it was possible to observe these structures and the individual droplets deposited on the ultra-thin layer of the sample. These individual droplets were oval shaped, with sizes similar to the values obtained by the LD instrument (F1 d50 ~99 nm, F2 d50 ~110 nm), but much higher than the PCS results (F1 Z-ave ~58 nm, F2 Z-ave ~56 nm). The shift from isotropic to anisotropic structures could be of practical interest in topical application, since liquid crystalline formulations are recognized to have prolonged hydrating effect due to their similarity with the skin lamellar structures [18,43]. The other LC structures (hexagonal, cubic) can also exhibit sustained release of actives or increased bioactivity, depending on the location of actives entrapped in their structure [45]. It should also be noted that the undiluted samples of the transient microemulsion phases could not be completely dried, which disabled the AFM analysis in our experimental setting. This was in line with the finding of non-freezable water during the DSC investigation, corresponding to the W/O microemulsion structure.

### 3.3. Screening of the Nanoemulsion Biological Activity

#### 3.3.1. Preparation and Stability of the Optimized Nanoemulsions

Red raspberry seed oil—RO, a natural oil derived from food industry byproducts, has been recognized as a rich source of bioactives, e.g., polyunsaturated fatty acids—PUFAs, and antioxidants—tocopherols, tocotrienols, carotenoids, polyphenol tyrosol, ellagic acid [1,2,3,4]. In our previous study [9], different types of ROs (cold-pressed vs. CO_2_-extracted, organic vs. non-organic, refined vs. unrefined) were thoroughly investigated. It was found that the organic, cold-pressed and unrefined oil containing 6.62% saturated fatty acids (C16:0 palmitic and C18:0 stearic acid) and 92% PUFAs (58% C18:2 ω6 linoleic, 22% C18:3 ω3 linolenic and 12% C18:1 ω9 oleic acid) was the most suitable for the preparation of stable Polysorbate 80-based nanoemulsions. Moreover, the Raman spectroscopic investigations of the ROs and RO-loaded nanoemulsions revealed that the oil’s fatty acid profile plays an important role in the formation and stability of nanoemulsions; it has also detected the differences in carotenoid content among the ROs [9].

**Figure 5:**
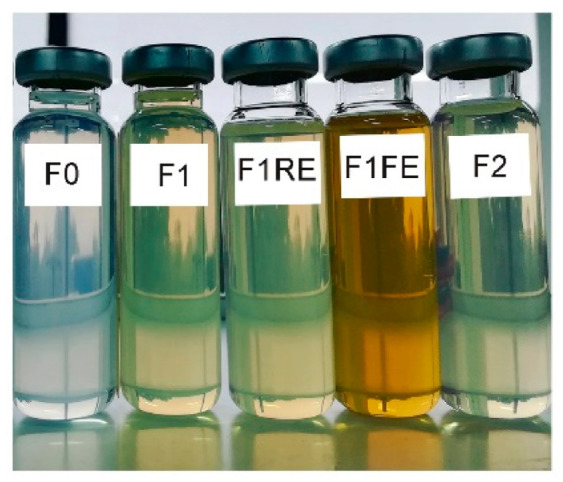
Visual appearance of the selected nanoemulsion samples prepared for in vitro and in vivo investigations: F0 (blank nanoemulsion without antioxidants), F1 (RO as lipophilic antioxidant, prepared with cosurfactant 1), F1 red raspberry (RE) and F1 French oak (FE) (with RO and additional hydrophilic antioxidants—RE, and FE, respectively, at 5 wt% relative to nanoemulsion total mass), and F2 (prepared with RO and cosurfactant 2, without additional hydrophilic antioxidants).

In this study, the optimal RO was incorporated in polyglycerol ester-based O/W nanoemulsion prepared with EP as a carrier oil. This resulted in a smaller droplet size and lower polydispersity in comparison to the blank nanoemulsion (blank: F0, Z-ave: 131.7 nm and PDI: 0.226, vs. RO-loaded nanoemulsions: F1 and F2, Z-ave < 60 nm and PDI ≤ 0.1), indicating a good system stability [39,46]. The compatibility of these RO-loaded polyglycerol ester-based nanoemulsions with hydro-glycolic antioxidant extracts from red raspberry (RE) and French oak fruit (FE) was also investigated, based on our previous findings that these additives can improve physicochemical stability and antioxidant/antiproliferative activity of Polysorbate 80-based nanoemulsion [9]. It was found that both RE and FE can be added at a maximum of 5 wt% of the nanoemulsion, but they had to be added after the phase transition, i.e., when the O/W nanoemulsion has already been formed. In this case, the addition of RE and FE extracts did not have any negative impact on nanoemulsion formation. The obtained nanoemulsions retained their semi-transparent appearance (Figure 5), due to very fine droplet size and uniform droplet size distribution (Z-ave < 60 nm, PDI ≤ 0.1, Table 2).

Since there were no signs of creaming, phase separation or dramatic increase in droplet size or PDI value, it was concluded that the optimized nanoemulsions showed satisfactory preliminary stability after 3 months of storage at room temperature, which are anticipated storage conditions for the nanoemulsions containing natural bioactive ingredients (Table 2). Small changes in the pH values and electrical conductivity that were observed during storage are in line with the previous reports regarding nanoemulsions containing natural plant oils [28,46]. This can be ascribed to the partial hydrolysis of the fatty acid esters in natural oils, and a possible “leakage” of some fatty acids from the surfactants based on polyglycerol esters.

#### 3.3.2. In Vitro Antioxidant Activity

The optimized formulations containing lipophilic antioxidants (from RO) with or without additional hydrophilic antioxidants (RE, FE) were checked for their ability to scavenge the DPPH free radical, as a fast screening procedure which can detect the antioxidant activity of the natural oils [47,48] and nanoemulsions [39,46] (Figure 5, Table 3). The DPPH assay allows evaluation of both lipophilic and hydrophilic substances [47], thus it was appropriate for the investigation of our nanoemulsion samples. The results presented in Table 3, revealed the concentration dependence of antioxidant activity for all nanoemulsions. The prominent synergistic effect between RO (containing carotenoids) and FE (containing polyphenols) resulted in very high percentage of free radical inhibition (>90% INH DPPH), while RE contributed only discretely to the overall activity. This was in line with our previous study involving Polysorbate 80-nanoemulsions, where the antioxidant activity of these pure raw materials was also reported [9]. Importantly, high antioxidant activity of polyglycerol-ester based nanoemulsions was preserved after 3 months of storage at room temperature.

#### 3.3.3. In Vitro Cytotoxic Activity

**Figure 6:**
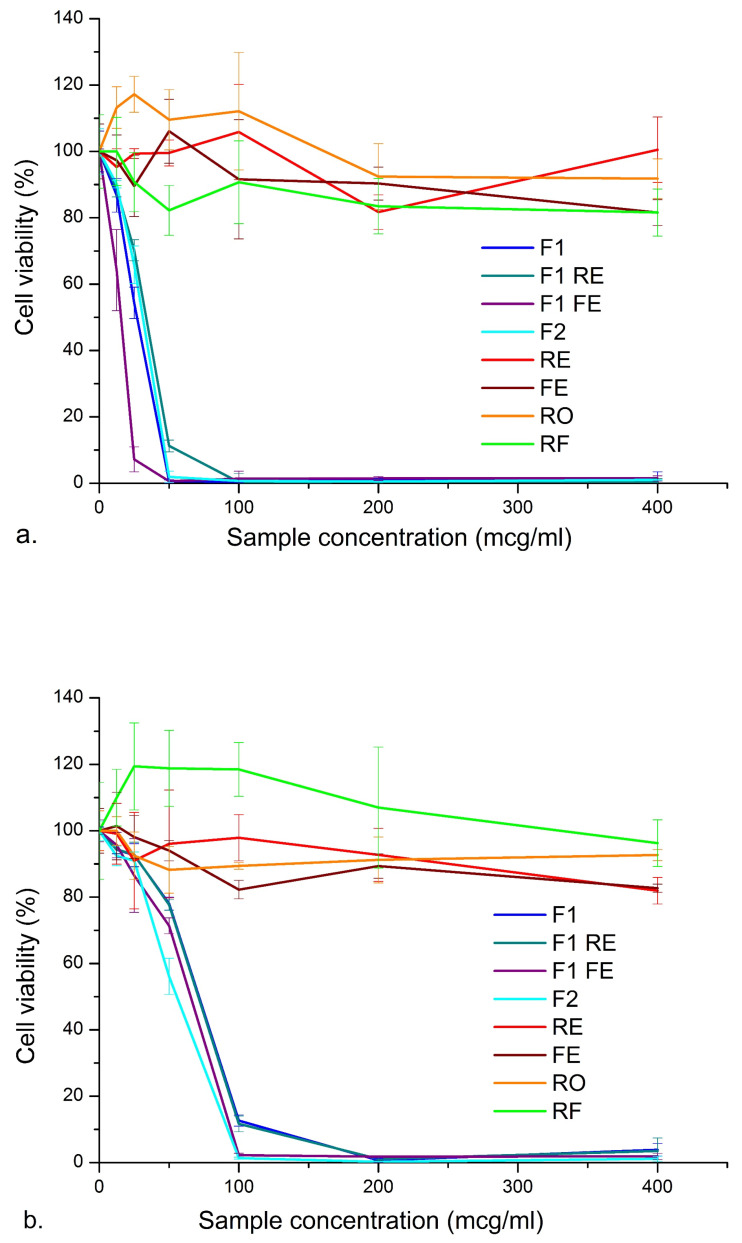

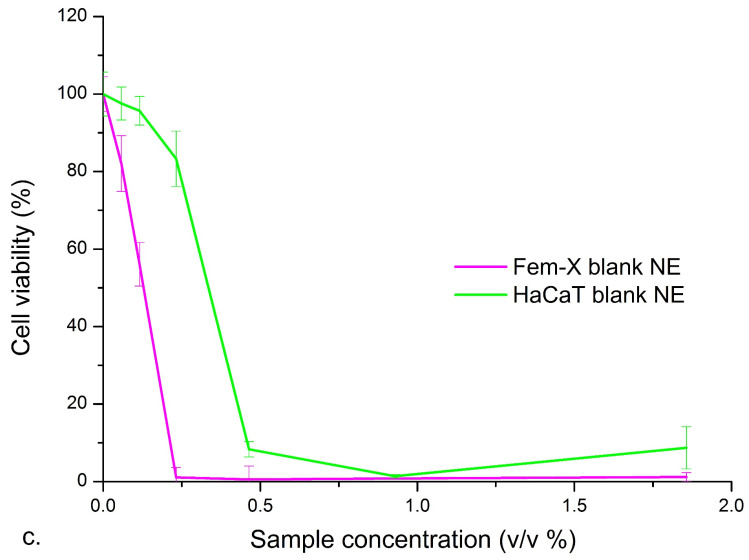
Cell viability percentage depending on the sample concentration: (**a**) effect of the raw materials and the corresponding nanoemulsions on malignant Fem-X cell line, (**b**) effect of raw materials and the corresponding nanoemulsions on normal HaCaT cells, (**c**) effect of blank nanoemulsions on malignant Fem-X and normal HaCaT cells. Each experiment was repeated three times and the results were presented as the mean value ± SD.

The natural raw materials used in this study (RO, RE, FE and RF) and the optimized nanoemulsions prepared with them were evaluated for their cytotoxic effect and selectivity to malignant melanoma (Fem-X cells) when compared to healthy human keratinocytes (HaCaT cells). None of the tested raw materials have shown antiproliferative effects on the selected cell lines, even at higher test concentrations than in our previous study (IC50 > 400 µg/mL, Table 4, Figure 6a,b, Figures S2 and S3, Supplementary file) [9]. However, the corresponding polyglycerol ester-based nanoemulsions showed prominent cytotoxic effect on the Fem-X cells (IC50 < 30 µg/mL), which was the opposite to our previous findings regarding Polysorbate 80-based nanoemulsions (IC50 > 200 µg/mL) [9]. In addition, much higher IC50 values were obtained for HaCaT cells (IC50 ~51 to 164 µg/mL), as shown in Table 4 and Figure 6b, confirming the selective cytotoxicity of the tested nanoemulsions to malignant Fem-X cells, which could be expressed as selectivity indices and selectivity scores [49,50]. Interestingly, the highest selectivity score (SI score 3) was observed for F1 formulation without hydrophilic additives, implying that the nanoemulsified RO was the main active ingredient responsible for such activity. It appears that the hydrophilic additives RE and FE and the fragrance/cosurfactant RF have increased antiproliferative effect on human keratinocytes, implying that their content may need to be reduced. However, these formulations are still classified as moderately selective (SI score 2), the same as the blank nanoemulsion containing EP (ethylhexyl pelargonate) without antioxidants (Figure 6c, Figure S4, Supplementary file). The antiproliferative activity of the blank nanoemulsion could be linked to the higher concentration range used in this screening, since it is known that higher concentrations of nanoemulsion stabilizers can induce toxicity to cultured cells due to their solubilizing effects [51], even though they are considered biocompatible. According to the previous reports [49,50], if the IC50 value is below 30 µg/mL for the tested plant material it can be considered a promising anticancer agent, which indicates that some of our materials are good candidates for further drug development, providing they are present in the form of nanoemulsions.

It should be noted that the 2D cell culture model used in this study has some limitations, not being an exact replica of the natural tissue structure. It does not always mimic the interactions between the cellular and extracellular environment, hence the changes in cell morphology, polarity and the method of cell division could also take place and lead to inaccurate conclusions [52].

### 3.4. In Vivo Safety and Efficacy Assessment

Despite the growing popularity of natural ingredients in cosmetic/pharmaceutical industry, the bio-origin must not be interpreted as an automatic provision of a safe or effective ingredient [17]. The polyglycerol-ester based surfactants employed in our study are naturally-derived and considered biocompatible, but due to their relatively high amount in the formulation and the addition of cosurfactants, it was necessary to assess their safety profile [40]. It should be noted that there are no reports involving in vivo studies regarding polyglycerol ester-based nanoemulsions and only a few in vivo studies involving natural seed oils or extracts incorporated in nanocarriers [12,28,35]. Therefore, a well-designed in vivo study on human volunteers should provide additional information on the safety of nanoformulations and a valuable insight into their efficacy upon topical application.

**Figure 7:**
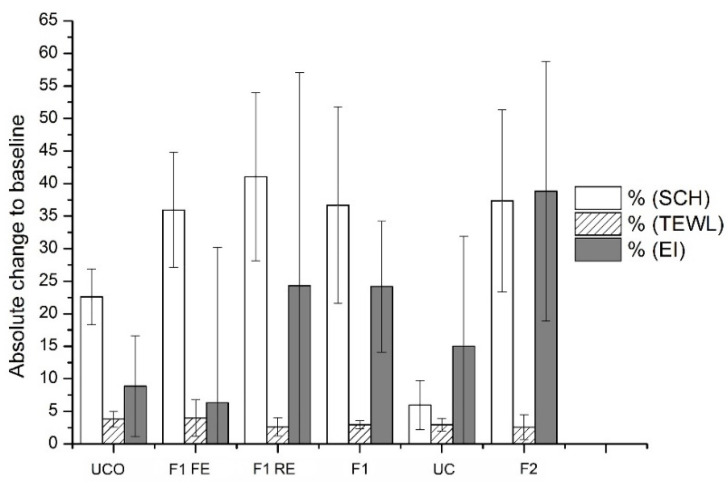
Skin irritation test—the influence of the investigated samples on in vivo measured skin parameters stratum corneum hydration (SCH), transepidermal water loss (TEWL), skin erythema index (EI) and both controls (under occlusion—UCO and without occlusion—UC); the results are shown as absolute changes of mean values on the second vs. first day, with the standard error of means; there were no statistically significant differences between the second vs. first day for any of investigated samples and controls.

In vivo skin irritation potential can be assessed by analyzing several parameters relevant to skin health and function (i.e., stratum corneum hydration—SCH, transepidermal water loss—TEWL, and skin erythema index—EI) after a single application of a particular formulation under a 24-h occlusion on the skin of healthy volunteers [40,53]. The results presented in Figure 7 confirmed the satisfactory safety profile of all investigated samples, since there were no instances of skin irritation, significant increase in TEWL or EI or decrease in SCH, which would indicate impaired skin barrier function [27,40].

**Figure 8:**
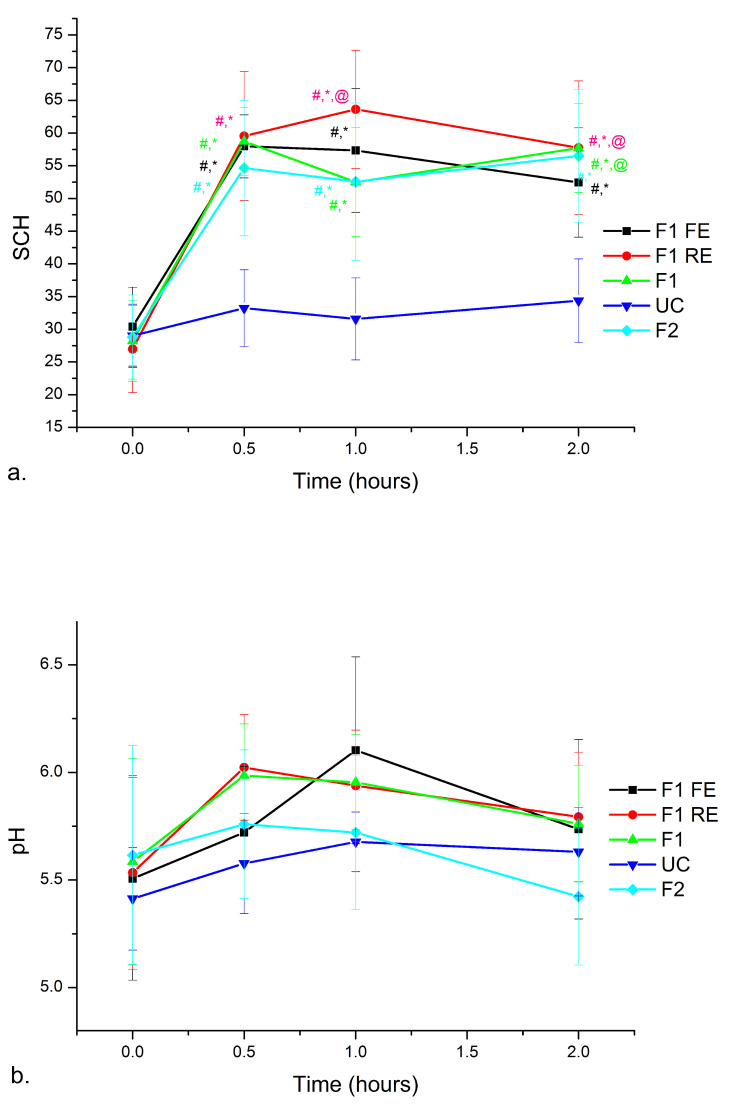
(**a**) The effects of topical application of the samples on SCH in a short-term study. Significant differences (*p* < 0.05) being marked with (#) for the difference to untreated control (UC), with (*) for the difference to baseline and with (@) for the difference to all other investigated samples; (**b**) skin pH values as a function of time (hours) after nanoemulsion application.

The skin hydration (moisturization) potential is a very important issue to be considered during the development of new topical formulations. Optimal skin hydration is reflected in a pleasant visual appearance (i.e., smoothness), indicating a good skin health and affecting skin permeability for topically applied drugs [12,54]. Therefore, an in vivo short-term study was conducted to assess stratum corneum water content after a single application without occlusion. It was found that the application of each nanoemulsion resulted in a significant improvement in the skin moisture level (SCH increase at 0.5, 1 and 2 h after application), compared to both untreated skin and baseline values (Figure 8a). It was also found that the skin pH value did not show any significant change during 2 h after application (Figure 8b), indicating that the formulations did not affect skin barrier function [12,28]. Therefore, our investigations have shown that the polyglyderol-ester based nanoemulsions loaded with red raspberry seed oil and hydro-glycolic fruit extracts from red raspberry or French oak fruit could be promoted as mild multifunctional topical preparations.

## 4. Conclusions

This study explores the potential of a novel group of naturally-derived surfactants, based on polyglycerol esters, in the production of topical nanoemulsion formulations. Specifically, polyglycerol-4 laurate was used as the main ingredient in the complex surfactant mix, in order to develop biocompatible nanoemulsion carriers suitable for natural seed oils and hydrophilic extracts. The PIC method performed at room temperature was chosen as an optimal low energy production process for the thermosensitive natural ingredients. The first stage of the process was the production of the blank nanoemulsion prepared with ethylhexyl pelargonate, a naturally-derived carrier oil novel to the nanoformulation process, in combination with the preservative mix based on phenoxyethanol or fragrance oil, both exhibiting a cosurfactant behavior. When red raspberry seed oil (RO) was added to the oil phase, an additional cosolvent (glycerol) was necessary in order to obtain nanoemulsion. It was found that nanoemulsions were formed by the dilution of the microemulsion transient phase, as confirmed by visual appearance, percentage of transmittance, microscopic, rheological and DSC investigations. The obtained basic RO-loaded formulation was further enriched with hydro-glycolic fruit extracts from red raspberry or French oak, while maintaining the semi-transparent appearance due to the small droplet sizes (Z-ave: 50 to 70 nm, PDI value ≤ 0.1) and satisfying stability during three months of storage at room temperature.

Since natural origin is not an equivalent to a safe and effective product, the study has included both in vitro screening of biological activity and in vivo efficacy and safety assessments of the nanoemulsions. The highest antioxidant activity (~92% inhibition of DPPH radical) was achieved with the formulation containing both lipophilic (RO) and hydrophilic antioxidants (FE), based on their synergistic effect. This high antioxidant activity was preserved after three months of storage at room temperature. The nanoemulsion carrier significantly increased the cytotoxic effect of tested raw materials, especially in the case of RO, towards malignant melanoma (Fem-X) cells when compared to normal human keratinocytes (HaCaT). In vivo study on human volunteers has implied a satisfactory safety profile and a significant improvement in skin hydration during 2 hours after application for all nanoemulsions. To conclude, polyglycerol ester-based nanoemulsions can be promoted as effective carriers for red raspberry seed oil and/or fruit extracts in topical formulations intended for skin protection and hydration.

## Figures and Tables

**Table 1:** Composition of the selected nanoformulations (transient phases and corresponding nanoemulsions), their percentage of transmittance (%TP) at 600 nm measured 24 h after preparation, and flow rheological parameters (n-flow behavior index, K-consistency index) measured 7 days after preparation.

Formulation Name	Water Phase wt% (Glycerol: Water)	Oil Phase Phase/Cosurfactant wt%	S Mix wt%	% TP	n-Index	K-Index
F0 30%	30 (1.5: 28.5)	35 (7 cosurfactant 2: 28 EP)	35	94.82 ± 0.005	0.9895 ± 0.0090	0.0532 ± 0.0026
F0 50%	50 (2.5: 47.5)	25 (5 cosurfactant 2: 20 EP)	25	87.57 ± 0.006	0.9828 ± 0.0026	0.0683 ± 0.0008
F0 80%	80 (4: 76)	10 (2 cosurfactant 2: 8 EP)	10	82.16 ± 0.037	1.0040 ± 0.0019	0.0045 ± 0.0004
F1 30%	30 (9: 21)	35 (1.75 cosurfactant 1: 7 RO: 26.25 EP)	35	94.42 ± 0.004	0.9843 ± 0.0102	0.0699 ± 0.0037
F1 50%	50 (15: 25)	25 (1.25 cosurfactant 1: 5 RO: 18.75 EP)	25	81.95 ± 0.002	0.9938 ± 0.0239	0.0617 ± 0.0073
F1 80%	80 (24: 56)	10 (0.5 cosurfactant 1: 2 RO: 7.5 EP)	10	76.18 ± 0.019	0.9582 ± 0.0136	0.0319 ± 0.0022
F2 74%	74 (22.2: 51.8)	13 (2.6 cosurfactant 2: 2.6 RO: 7.8 EP)	13	71.12 ± 0.047	0.9894 ± 0.0178	0.0532 ± 0.0056
F2 80%	80 (24: 56)	10 (2 cosurfactant 2: 2 RO: 6 EP)	10	78.10 ± 0.016	0.9876 ± 0.0133	0.0073 ± 0.0004

**Table 2:** Z-average droplet size (Z-ave) and polydispersity index (PDI) of the selected nanoemulsion samples, pH and electrical conductivity values, measured 24 h after preparation and after 3 months of storage at room temperature.

Sample Name	Z-Ave (nm)	PDI	pH	El. Cond.
				(µS/cm)
F0	24h:131.70 ± 1.114	24h: 0.226 ± 0.012	24h: 4.56 ± 0.02	24h: 128.6 ± 0.20
	3m: 138.30 ± 1.358	3m: 0.162 ± 0.023	3m: 4.29 ± 0.05	3m: 145.4 ± 0.96
F1	24h: 58.21 ± 5.187	24h: 0.071 ± 0.015	24h: 4.53 ± 0.03	24h: 30.68 ± 1.66
	3m: 60.12 ± 8.831	3m: 0.099 ± 0.037	3m: 4.15 ± 0.01	3m: 48.50 ± 0.40
F1 RE	24h: 55.48 ± 6.769	24h: 0.071 ± 0.010	24h: 4.00 ± 0.04	24h: 42.73 ± 0.35
	3m: 62.73 ± 1.255	3m: 0.079 ± 0.007	3m: 3.83 ± 0.01	3m: 52.63 ± 0.42
F1 FE	24h: 56.37 ± 5.130	24h: 0.058 ± 0.024	24h: 4.48 ± 0.07	24h: 117.53 ± 0.06
	3m: 59.21 ± 10.09	3m: 0.089 ± 0.005	3m: 4.47 ± 0.15	3m: 124.87 ± 0.21
F2	24h: 55.62 ± 1.164	24h: 0.093 ± 0.022	24h: 4.49 ± 0.28	24h: 35.11 ± 4.99
	3m: 59.77 ± 2.208	3m: 0.098 ± 0.011	3m: 4.29 ± 0.03	3m: 41.53 ± 4.46

**Table 3:** Antioxidant activity expressed as a percentage of inhibition (%INH) of DPPH free radical of the selected nanoemulsions at different concentrations 24 h after preparation, and after 3 months of storage at room temperature.

Sample Name	% INH DPPH			% INH DPPH
	24 h After Preparation			After 3 Months
	10 µL/mL	20 µL/mL	30 µL/mL	30 µL/mL
F0	0.18 ± 0.03	0.55 ± 0.04	0.70 ± 0.07	1.2 ± 0.15
F1	3.73 ± 0.10	6.41 ± 0.14	8.37 ± 0.55	10.19 ± 0.62
F1 RE	3.91 ± 0.09	7.33 ± 0.12	9.53 ± 0.41	11.74 ± 0.47
F1 FE	90.87 ± 0.31	92.49 ± 0.29	91.65 ± 0.93	93.79 ± 0.11
F2	3.67 ± 0.06	8.37 ± 0.07	9.90 ± 0.56	10.98 ± 0.27

**Table 4:** In vitro cytotoxic activity (IC50 values), the selectivity indices and the SI scores of the raw materials and the optimized nanoemulsions tested against the tumor cell line (Fem-X) and the non-tumor cell line (HaCaT).

Sample Name	Fem-X	HaCaT	SI *	SI Score **
	IC50 (µg/mL)	IC50 (µg/mL)		
F1	31.41 ± 8.75	164.16 ± 34.57	5.23	3
F1 RE	28.99 ± 4.99	93.79 ± 42.55	3.24	2
F1 FE	25.06 ± 6.58	65.06 ± 8.14	2.60	2
F2	29.41 ± 2.52	51.05 ± 6.41	1.74	2
RE	>400	>400	NV	NV
FE	>400	>400	NV	NV
RO	>400	>400	NV	NV
RF	>400	>400	NV	NV

* SI index = The selectivity index is the ratio of the IC50 values of the samples on HaCaT cells to those in the Fem-X cell lines. ** SI score: score 1 (unselective) = SI ≤ 1, score 2 (moderately selective) = 1 < SI < 5, score 3 (selective) = SI ≥ 5. NV–no value, since IC50 was not obtained at concentrations of ≤400 µg/mL.

## Data Availability

Data is contained within the article or supplementary material. The data presented in this study are available in [https://doi.org/10.3390/nano11010217].

## Supplementary Materials

The following are available online at https://www.mdpi.com/2079-4991/11/1/217/s1, Table S1. Z-average droplet size (Z-ave) and polydispersity index (PDI) of blank nanoemulsions, nanoemulsions prepared with cosurfactant 1 (phenoxyethanol, ethylhexyl glycerol) and/or cosurfactant 2 (raspberry fragrance—RF); Table S2. Z-average droplet size (Z-ave) and polydispersity index (PDI) of the red raspberry seed oil (RO)-loaded nanoemulsions with additional hydrophilic antioxidant extracts from red raspberry—RE or French oak—FE fruit in the nanoemulsion water phase; Figure S1. Representative flow curves of F1 formulations; Figure S2. Optical density at 570 nm of normal human keratinocytes (HaCaT cells); Figure S3. Optical density at 570 nm of Fem-X human malignant melanoma cells; Figure S4. Optical density at 570 nm of the blank nanoemulsion on HaCaT and Fem-X cells.
