# Supplementary material for: Polyglycerol Ester-Based Low Energy Nanoemulsions with Red Raspberry Seed Oil and Fruit Extracts: Formulation Development toward Effective In Vitro/In Vivo Bioperformance
Source: Nanomaterials (Basel). 2021 Jan 15;11(1):217. doi: 10.3390/nano11010217 (PMC7830947; doi:10.3390/nano11010217)
Supplement: Supplementary file 1 [file nanomaterials-11-00217-s001.pdf]

Supplementary file

Polyglycerol Ester-Based Low Energy Nanoemulsions with Red Raspberry Seed Oil and Fruit Extracts: Formulation Development toward Effective In vitro/In vivo Bioperformance

Formulation optimization - influence of the cosurfactants, oil and water phase composition

Table S1. Z-average droplet size (Z-ave) and polydispersity index (PDI) of blank nanoemulsions prepared with cosurfactant 1 (phenoxyethanol, ethylhexyl glycerol) and/or cosurfactant 2 (raspberry fragrance). Each nanoemulsion contained 10 wt% polyglycerol ester-based surfactant mix, 10 wt% oil phase (ethylhexyl pelargonate + cosurfactant), and 80 wt% water. The samples were stored at room temperature, and the measurements were taken 24 hours and one month after preparation.

	**Z-ave (nm) 24 h**	**Z-ave (nm) 1 m**	**PDI 24 h**	**PDI 1 m**
**Cosurfactant 1**				
0.5 wt%	131.700 ± 1.114	157.167 ± 2.060	0.226 ± 0.012	0.201 ± 0.004
1 wt%	231.333 ± 5.525	2F, unstable	0.361 ± 0.031	2F, unstable
**Cosurfactant 2**				
0.5 wt%	89.760 ± 0.428	99.393 ± 0.840	0.112 ± 0.003	0.116 ± 0.010
1 wt%	86.397 ± 0.708	103.167 ± 0.814	0.116 ± 0.016	0.141 ± 0.008
1.5 wt%	89.627 ± 0.085	104.100 ± 1.153	0.078 ± 0.016	0.120 ± 0.019
2 wt%	89.563 ± 0.533	90.917 ± 0.293	0.069 ± 0.019	0.098 ± 0.006
2.5 wt%	85.153 ± 0.345	100.050 ± 1.516	0.099 ± 0.018	0.145 ± 0.021
3 wt%	92.443 ± 0.725	109.633 ± 1.002	0.122 ± 0.009	0.133 ± 0.019
3.5 wt%	101.100 ± 0.458	110.733 ± 1.159	0.105 ± 0.006	0.168 ± 0.012
4 wt%	161.700 ± 5.810	159.667 ± 5.372	0.314 ± 0.037	0.360 ± 0.061
**Cosurfactant mix**				
cosurfactant 1, 0.5 wt + cosurfactant 2, 2 wt%	216.000 ± 1.473	2F, unstable	0.398 ± 0.003	2F, unstable
cosurfactant 1, 1 wt% + cosurfactant 2, 2 wt%	328.410 ± 18.28	2F, unstable	0.629 ± 0.116	2F, unstable
cosurfactant 1, 1 wt% + cosurfactant 2, 1 wt%	255.212 ± 1.153	2F, unstable	0.272 ± 0.006	2F, unstable

2F –two phase system, i.e. phase separation in unstable formulations

Table S2. Z-average droplet size (Z-ave) and polydispersity index (PDI) of the red raspberry seed oil (RO)-loaded nanoemulsions with additional hydrophilic antioxidant extracts from red raspberry – RE or French oak – FE fruit in the nanoemulsion water phase. Each nanoemulsion contained 10 wt% polyglycerol ester-based surfactant mix, 10 wt% oil phase, 0 to 5 wt% hydrophilic extract and water phase (30 wt% glycerol in water), up to 80 wt%, relative to the nanoemulsion total mass. The formulations were stored at room temperature, and the measurements were taken 24 hours and three months after preparation.

	**Z-ave (nm) 24 h**	**Z-ave (nm) 2 m**	**PDI 24 h**	**PDI 2 m**
***F1** basic formulation **oil phase** contains: 2 wt% RO (lipophilic antioxidant), 0.5 wt% cosurfactant 1, 7.5 wt% EP* **Water phase**:				
NO hydrophilic antioxidant	64.328 ± 6.111	65.580 ± 1.074	0.063 ± 0.020	0.059 ± 0.008
1 wt% RE	55.150 ± 0.710	63.747 ± 0.819	0.073 ± 0.012	0.071 ± 0.049
2 wt% RE	54.917 ± 0.195	63.883 ± 1.115	0.074 ± 0.025	0.055 ± 0.006
3 wt% RE	57.243 ± 0.245	64.040 ± 1.713	0.066 ± 0.112	0.056 ± 0.003
4 wt% RE	62.633 ± 1.148	59.963 ± 0.963	0.049 ± 0.004	0.049 ± 0.021
5 wt% RE	59.350 ± 0.850	66.967 ± 1.662	0.066 ± 0.013	0.061 ± 0.019
1 wt% FE	52.237 ± 0.386	63.467 ± 1.290	0.082 ± 0.014	0.042 ± 0.019

2 wt% FE	53.187 ± 0.176	62.280 ± 0.951	0.097 ± 0.006	0.041 ± 0.018
3 wt% FE	67.587 ± 1.192	65.743 ± 1.401	0.044 ± 0.012	0.060 ± 0.006
4 wt% FE	66.630 ± 1.414	66.963 ± 1.589	0.063 ± 0.019	0.034 ± 0.027
5 wt% FE	68.770 ± 2.192	62.400 ± 0.607	0.054 ± 0.012	0.054 ± 0.009
F2 *basic formulation* ***oil phase*** *contains: 2 wt% RO (lipophilic antioxidant), 2 wt% cosurfactant 2, 6 wt% EP* ***Water phase:***				
NO hydrophilic antioxidant	58.980 ± 1.976	62.272 ± 4.054	0.079 ± 0.012	0.111 ± 0.016
1 wt% RE	56.543 ± 0.087	56.657 ± 1.583	0.076 ± 0.010	0.081 ± 0.009
2 wt% RE	52.853 ± 0.237	56.133 ± 1.793	0.119 ± 0.010	0.080 ± 0.015
3 wt% RE	53.030 ± 0.344	58.490 ± 1.032	0.098 ± 0.022	0.077 ± 0.016
4 wt% RE	54.100 ± 0.674	53.933 ± 1.851	0.072 ± 0.009	0.037 ± 0.011
5 wt% RE	56.700 ± 0.292	56.147 ± 1.925	0.109 ± 0.019	0.046 ± 0.014
1 wt% FE	64.923 ± 1.760	67.557 ± 1.819	0.059 ± 0.012	0.060 ± 0.014
2 wt% FE	55.560 ± 0.967	62.510 ± 1.886	0.063 ± 0.019	0.062 ± 0.021
3 wt% FE	63.907 ± 0.942	67.980 ± 1.763	0.044 ± 0.019	0.052 ± 0.006
4 wt% FE	64.697 ± 3.206	63.157 ± 1.783	0.042 ± 0.021	0.067 ± 0.013
5 wt% FE	57.373 ± 2.037	61.880 ± 2.654	0.056 ± 0.014	0.089 ± 0.017

Flow rheological measurements

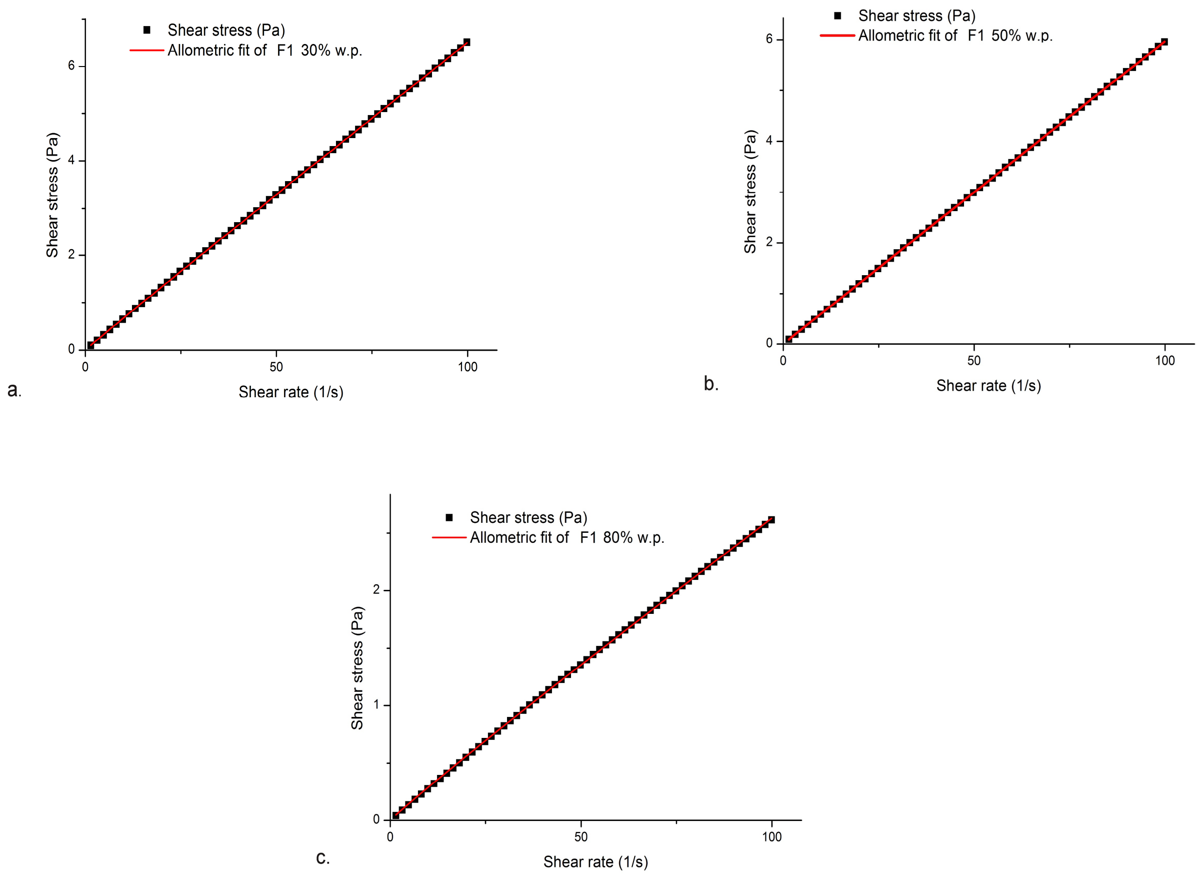

Figure S1. Representative flow curves of F1 formulations prepared with: (a) 30 wt% water phase (microemulsion phase) and low-energy nanoemulsions prepared with: (b) 50 wt% water phase and (c) 80 wt% water phase (30 wt% glycerin in water). The figures represent Newtonian flow behavior in all tested samples.

In vitro cell viability assay – optical density measurements

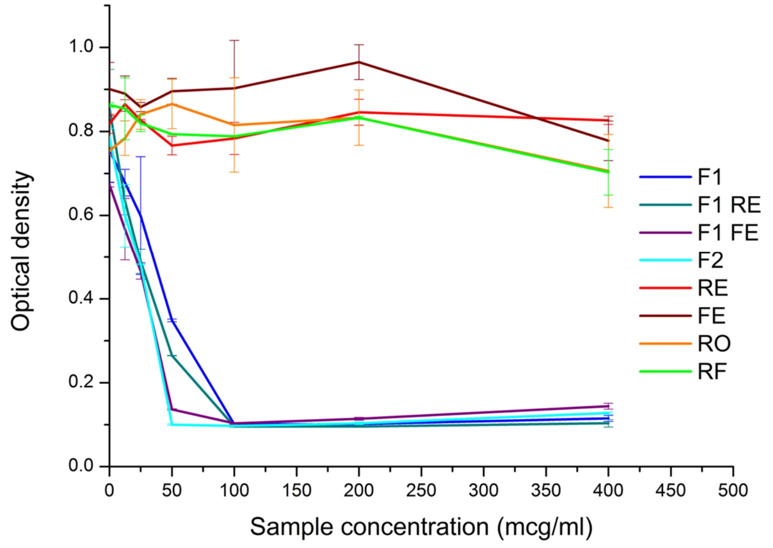

Figure S2. Optical density at 570 nm of normal human keratinocytes (HaCaT cells): effect of the raw materials and the corresponding nanoemulsions. Obtained data represent optical density of the cell culture depending on the sample concentration. Each experiment was repeated three times and the results were presented as the mean value ± SD.

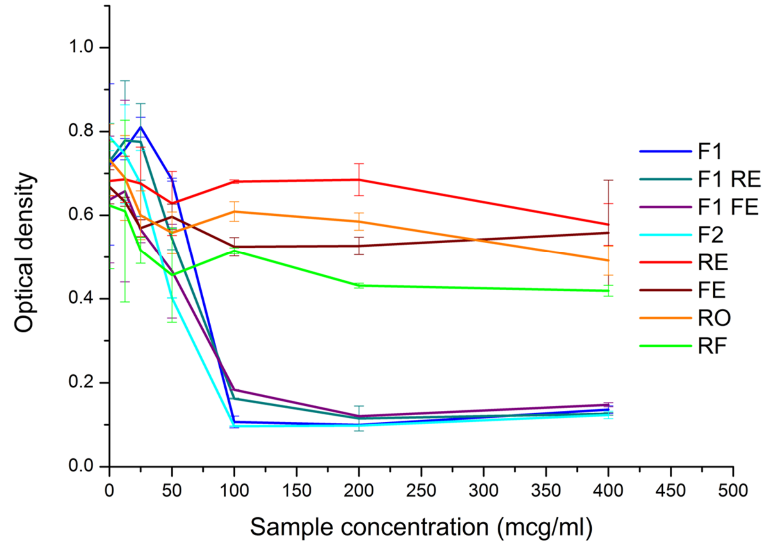

Figure S3. Optical density at 570 nm of Fem-X human malignant melanoma cells: effect of the raw materials and the corresponding nanoemulsions. Obtained data represent optical density of the cell culture depending on the sample concentration. Each experiment was repeated three times and the results were presented as the mean value ± SD.

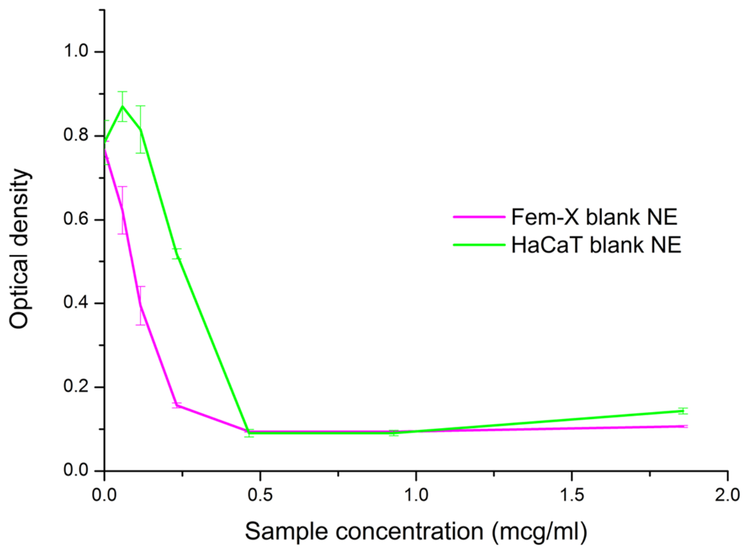

Figure S4. Optical density at 570 nm of the blank nanoemulsion on HaCaT and Fem-X cells. Obtained data represent optical density of the cell culture depending on the sample concentration. Each experiment was repeated three times and the results were presented as the mean value ± SD.
